# Adequacy of Severe Malaria Markers and Prognostic Scores in an Intensive Care Unit in Luanda, Angola: A Clinical Study

**DOI:** 10.3390/jcm9123862

**Published:** 2020-11-27

**Authors:** Maria Lina Antunes, Jorge Seixas, Humberto E. Ferreira, Marcelo Sousa Silva

**Affiliations:** 1 Hospital Américo Boavida, Luanda 160963, Angola; 2 Global Health and Tropical Medicine R&D Center, NOVA University, 1349-008 Lisbon, Portugal; jseixas@ihmt.unl.pt; 3 Institute of Hygiene and Tropical Medicine, NOVA University, 1349-008 Lisbon, Portugal; 4 CERENA, Instituto Superior Técnico, University of Lisbon, 1349-008 Lisbon, Portugal; hecsf@ff.ul.pt; 5 Departamento de Química Farmacêutica e Terapêutica (DQFT), Faculdade de Farmácia, Universidade de Lisbon, 1349-008 Lisbon, Portugal; 6 Departamento de Análises Clínicas e Toxicológicas, Centro de Ciências da Saúde, Universidade Federal do Rio Grande do Norte, Natal 59000-000, Brazil

**Keywords:** severe malaria, *Plasmodium falciparum*, SOFA score, intensive care unit, Angola

## Abstract

Severe *Plasmodium falciparum* malaria remains the primary cause of mortality in several African countries, including Angola, where severe malaria patient admission into intensive care units (ICU) is mandatory. The present observational and prospective study enrolled 101 consecutive severe malaria patients admitted at the ICU of Américo Boavida University Hospital (Luanda, Angola). Malaria was confirmed by microscopy and RDT, and WHO criteria were used to define severe malaria. The Sequential Organ Failure Assessment (SOFA) score was used to monitor organ dysfunctions. Surviving and nonsurviving patients were compared using bivariate statistical methods. Two-step cluster analysis was used to find discriminant organ dysfunctions that may correlate better with the observed mortality (16.8%), which was much lower than the one generated by the SOFA score. The study population was young, and 87% of the patients were local native residents. There was no statistically significant correlation between the parasitemia and the outcome. Hematological and cerebral dysfunctions were prevalent but were not discriminant when cluster analyses were performed to detect homogeneous subgroups of patients. In conclusion, the SOFA score was readily applicable and efficient in monitoring daily organ dysfunction but was not effective enough in predicting the outcome of severe malaria patients.

## 1. Introduction

Malaria is an infectious disease that is prevalent in several tropical and subtropical countries, representing a serious international public health problem. The etiological agent of malaria is a protozoan parasite of the genus *Plasmodium* and the main species of importance in human infection are *P. falciparum*, *P. vivax*, *P. ovale*, *P. malariae*, and *P. knowlesi. P. falciparum* is the most prevalent malaria parasite worldwide; it is responsible for the vast majority of severe-malaria-associated deaths [1].

Significant progress in the fight against malaria has been obtained in the last 20 years, with the incidence rate declining globally between 2010 and 2018 from 71 to 57 cases per 1000 population at risk. Nevertheless, according to WHO, an estimated 228 million cases of malaria still occurred worldwide in 2018, 93% of them in the African region. An estimated 405,000 deaths from malaria occurred globally in 2018, compared with 585,000 in 2010. A significant percentage of those deaths are concentrated in Africa: Nigeria accounted for almost 24% of all global malaria deaths, followed by the Democratic Republic of the Congo (11%), the United Republic of Tanzania (5%), and Angola, Mozambique, and Niger (4% each) (WHO World Malaria Report 2019). Thus, *P. falciparum* malaria remains the leading cause of mortality by a parasitic infection worldwide, especially in sub-Saharan Africa. In Angola, malaria is endemic in most provinces and constitutes the most notified disease and the principal cause of mortality in the country [2].

Immunity acquisition against malaria progresses slowly and, to be maintained, requires repeated exposure to a variety of *Plasmodium* antigens and maturation of the immune system with age [3]. Immunity against malaria is partial, species-specific, and labile. This semi-immunity allows infection without disease, may limit disease severity, and, in a context of stable *Plasmodium* transmission, constitutes the main reason for the decrease in incidences of severe malaria with age. Pathophysiologic and pathologic features of severe *P. falciparum* malaria include sequestration, cytoadherence, rosette formation, and reduced erythrocyte deformability. The ultimate consequence of this phenomenon is tissue hypoxia and organ dysfunction. Parasites can be found in the microvasculature of critical organs of the host, which reflects the systemic nature of *P. falciparum* malaria [4].

In severe malaria, different organs and systems are simultaneously affected, and if the infection is not efficiently treated, it progresses to multiorgan failure. Proper markers for malaria severity should, therefore, evaluate the functionality of all the affected organs and systems to find early prognostic factors. World Health Organization (WHO) criteria for severe malaria have been adapted for tropical regions, but, nevertheless, patients with single organ failure are weighed on equal terms as those presenting multiorgan failure [5].

Angola’s guidelines for malaria indicate that patients with severe disease criteria should be managed in intensive care units (ICUs). In the context of ICUs, correctly establishing the prognosis for a given patient is essential for individual prediction of diagnostic or therapeutic courses of action, as well as for optimized management of the frequently limited resources of this kind of service while maintaining quality of care, especially in low- and middle-income countries such as Angola. The Sequential Organ Failure Assessment (SOFA) score, which incorporates scores related to respiratory, cardiovascular, hepatic, hematological, renal, and neurological systems, is widely used in the ICU context for establishing the prognosis in severely ill patients [6].

The present study is designed to characterize the clinical and parasitological parameters of patients with severe malaria managed in an ICU facility in Luanda, Angola, and to clarify the adequacy of disease-severity scoring systems, such as SOFA, in correctly evaluating severe malaria patient prognosis in order to obtain better markers for mortality for this form of the disease.

## 2. Methodology

Between March 2011 and March 2013, an observational and prospective study was performed among patients hospitalized at the University Américo Boavida Hospital ICU, Luanda, Angola. This ICU admits patients ≥10 years of age. Inclusion criteria were (i) malaria diagnosis, confirmed by thick blood smear microscopy (Giemsa stain) and/or a rapid diagnostic test (RDT); (ii) symptoms and signs of severe malaria according to WHO classification; (iii) failure of two or more organ systems. Exclusion criteria were (i) pregnant women; (ii) patients with chronic comorbidities; (iii) other acute comorbidities that might result in organ failure, detected by differential diagnostic methods.

Patients were characterized by gender, age, comorbidity, and residence during the previous six months. All patients were managed according to the ICU’s protocol for malaria, which included an intravenous quinine (20 mg/kg) loading dose, followed by 10 mg/kg every 8 h for seven days. Clindamycin 200 mg/Kg was also administered twice a day for five days.

The clinical scenario was evaluated daily by the SOFA score. Each organ system was evaluated using a score ranging from zero to four according to the worst daily physiological and laboratory parameters, and six organ systems were studied, producing a final score ranging from 0 (best) to 24 (worst). Average SOFA was calculated using the first three days of results. SOFAmax and deltaSOFA, the difference between worst and better values of the first three days, were also calculated. These two SOFA indicators were used to calculate expected mortality rates based on the probability of death.

All data were obtained from the patient’s files using a structured questionnaire and introduced into an MS-Excel database. The SOFA score death probability values were directly calculated using the software available for this purpose at http://clincalc.com/icumortality/sofa.aspx. The database was double-checked before exporting it to SPSS-21 (IBM, NYC, USA). All statistical analyses were performed using this software (SPSS-21, IBM, NYC, USA) and included a bivariate analysis after the initial exploratory data analysis. Finally, the two-step cluster analysis algorithm (SPSS 21) was used to detect homogeneous subgroups of patients that were discriminated according to the pattern of organ dysfunction observed. The detected subgroups/clusters were further used to correlate the observed mortality with the one expected/predicted by the SOFA score.

Informed consent was obtained from all patients and/or relatives. The study was approved by Angola’s Ministry of Health (MINSA) Ethical Committee and by the hospital’s Directorate (Protocol number 4-2012-PN of 22 February 2012).

## 3. Results

The study population (*n* = 101) was predominantly male (61.4%), with a mean age of 21 years (standard deviation 10.72). The majority of the patients had no comorbidities (72.2%), and 87% were native residents in Luanda and considered semi-immune to malaria. There were 17 deaths (16.8%) during the present study. No statistically significant differences in sociodemographic characteristics of the population were detected between surviving and nonsurviving patient groups. The mean duration of ICU total stay was 6.1 days. The mean ICU stay for surviving patients (6.62 ± 0.404 days, mean ± SE) was significantly higher than for nonsurviving (3.65 ± 0.732 days) patients (one-way ANOVA, *p* = 0.002).

Despite the severe clinical presentation, 57.4% of the study patients had ≤50,000 parasites (trophozoites per μL) in blood smears upon admission (Table 1). There was no statistically significant difference between mean parasite density (parasite per μL) in blood smears vis-a-vis the outcome (mean ± SE; surviving 69,313 ± 10,267, nonsurviving 66,912 ± 214,120; one-way ANOVA, *p* = 0.923).

The laboratory data of patients surviving and not-surviving the malaria episode are shown in Table 2. All mean values were significantly different between the two groups except for platelet count (ANOVA, *p* = 0.174) and serum total bilirubin values (ANOVA, *p* = 0.424), for which the nonsurviving group had generally worse values than the surviving patients.

**Figure 1:**
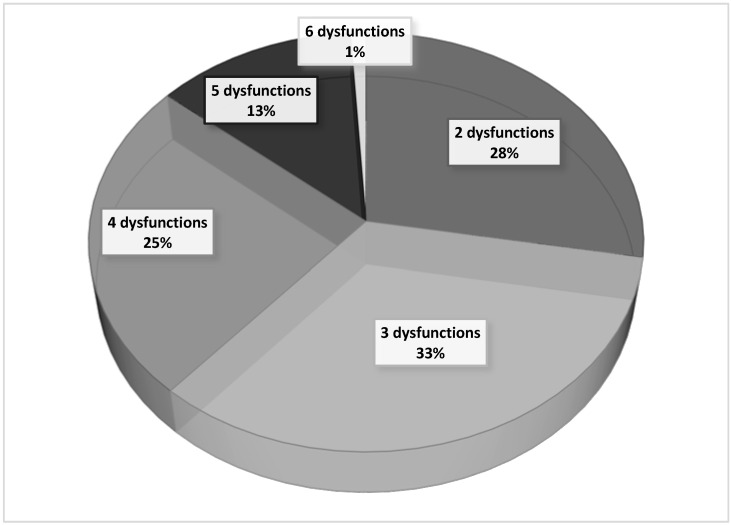
Relative distribution of the number of organ dysfunctions in hospitalized patients with severe malaria.

To determine the SOFA score, the worst daily values of laboratory data from patients with severe malaria were used. During the first three days of hospitalization, more than two organ dysfunctions were recorded in 72% of patients with severe malaria. On the other hand, four or more organic disorders were simultaneously present in 39% of these patients, confirming the severity of the clinical cases included in the present study. These data are shown in Figure 1.

**Figure 2:**
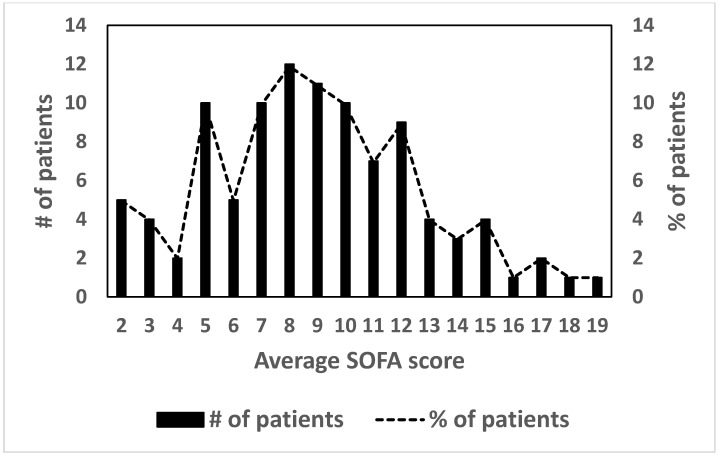
Distribution of patients with severe malaria according to the average Sequential Organ Failure Assessment (SOFA) score of the population studied (*n* = 101).

Regarding the distribution of the average SOFA score in the studied population, the observed value is 8.92 ± 3.81 (mean ± SD). Figure 2 shows the distribution of the SOFA score of patients hospitalized with severe malaria. Considering a score value equal to 8.0 as the cut-off point, it is observed that 64.3% (*n* = 65) of the patients had a high (>50%) probability of individual death, as predicted by the SOFA score.

Regarding the need for support and invasive intervention techniques in the population with severe malaria, it is observed that the majority of patients did not require life-supporting invasive techniques (59.4%). Hemodialysis alone was used in 14.8% of patients and invasive mechanical ventilation alone was needed in 10.8% of the patients; the combined use of hemodialysis and invasive ventilation was applied in 9.8% of the patients (Table 3).

Variables related to the severity of the patient clinical status and the use of life-support interventions for individuals hospitalized with severe malaria are presented in Table 4. All these variables, obtained during the first three days of hospitalization, showed very significant statistical differences between surviving and nonsurviving groups except for the use of amine vasoactive treatment to maintain adequate blood pressure (χ^2^, *p* = 0.656). This treatment was only applied to 4 patients included in the study, a very small sample size that may justify the nondetection of a significant statistical difference. In general, the nonsurviving group had worse mean values than the surviving patients. However, it is worth mentioning that the numbers of life support interventions applied were higher in surviving patients, with an extremely significant difference from the nonsurviving group (χ^2^, *p* < 0.001).

**Figure 3:**
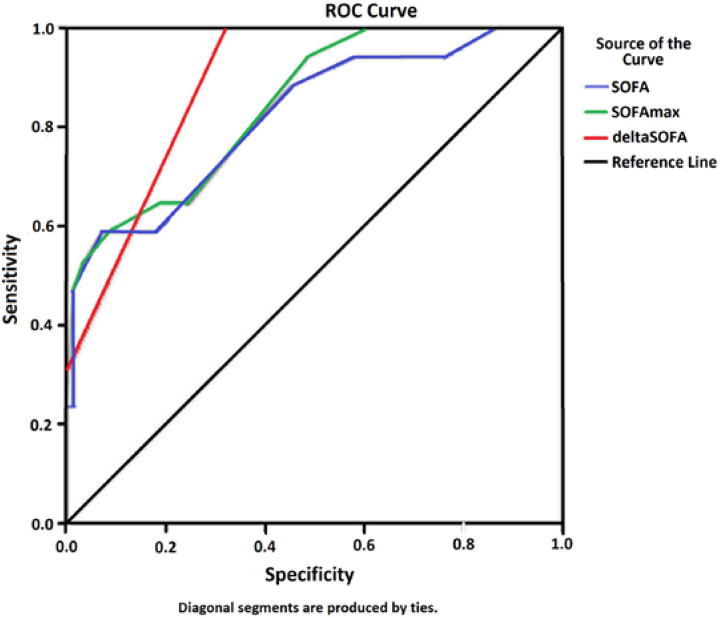
ROC curves for three variants of the SOFA score based on mortality observed in hospitalized patients (*n* = 101) with severe malaria.

The overall mortality rate observed in patients with severe malaria was 16.8%, lower than that predicted by the average scores of SOFA and SOFAmax. Figure 3 shows that the ROC curves for these two variants of the SOFA score were significant and similar (mean SOFA AUC 0.820, SOFAmax AUC 0.845; 95% CI). Figure 3 also includes a third variant of SOFA that shows better sensitivity (deltaSOFA AUC 0.887; 95% CI).

**Figure 4:**
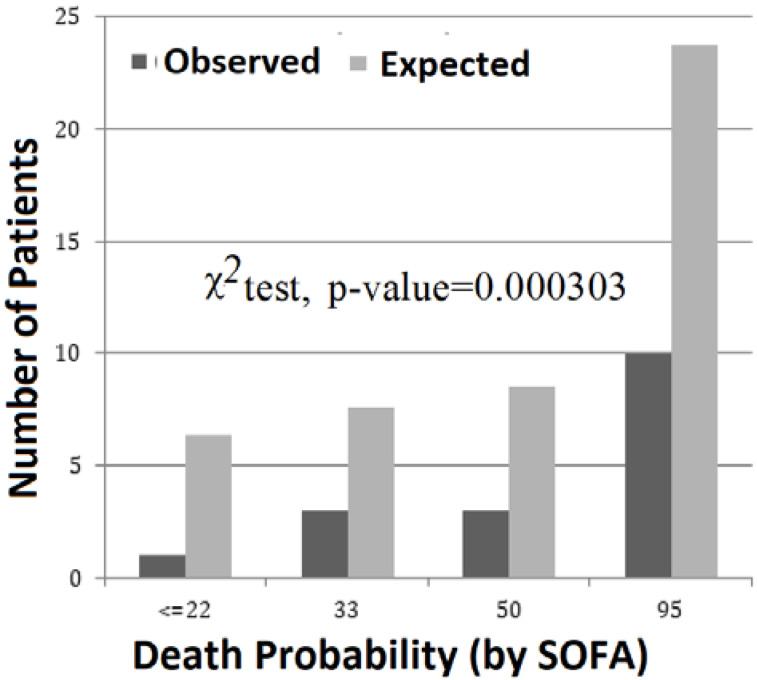
Comparison between the number of deaths in patients with severe malaria and the number of deaths expected/predicted by SOFA score.

In reality, the mortality observed in patients with severe malaria is much lower than the number of expected mortality predicted by the SOFA score. Figure 4 shows the distribution of observed deaths versus expected deaths predicted by the average SOFA score, estimated by the values of probability of death calculated directly using the calculator available at http://clincalc.com/icumortality/sofa.aspx. The difference between observed and expected deaths was greater in the most critical patients (χ^2^, *p* <0.001) included in the group, with a 95% probability of death, calculated from the average SOFA score.

**Figure 5:**
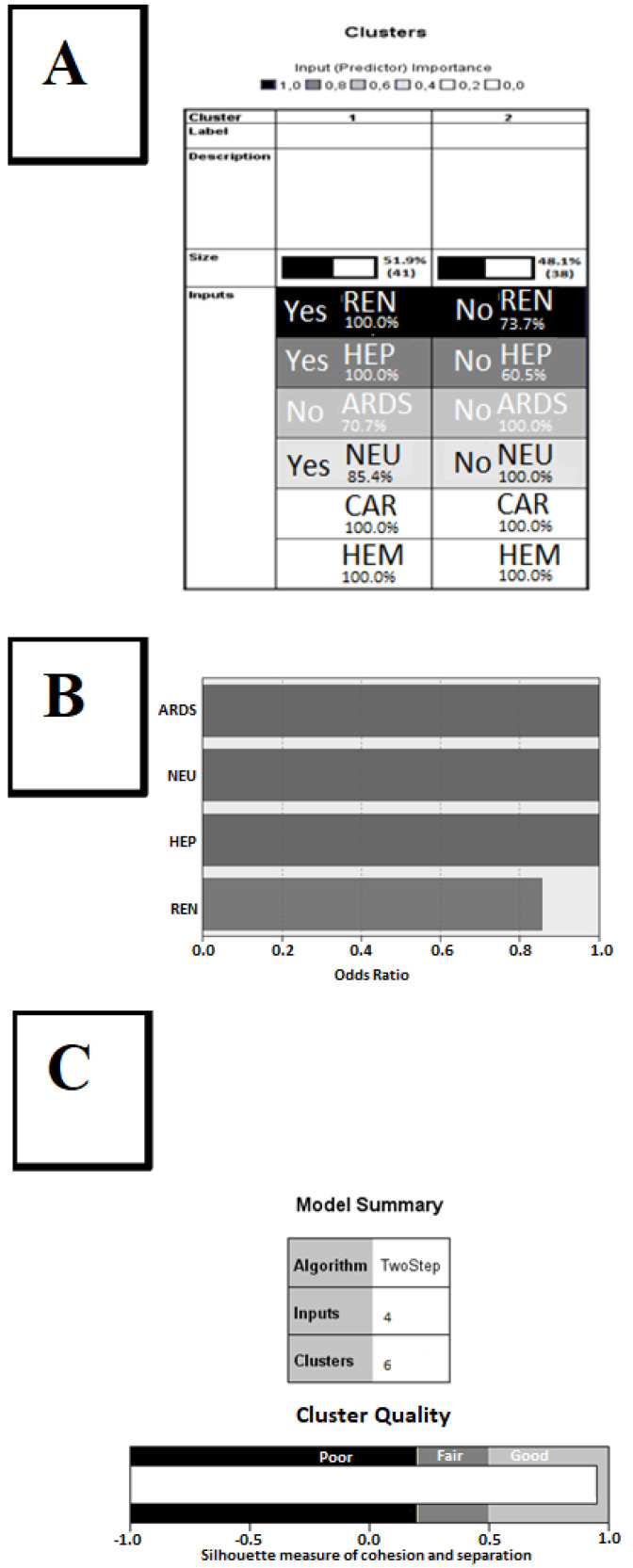
Cluster analysis of organ dysfunctions. (**A**) Graphic output of two-step cluster analysis based on 6 organ dysfunctions (*n* = 101) used to define the SOFA score. Organ dysfunctions mentioned: REN = renal, HEP = hepatic, ARDS = respiratory distress, NEU = neurologic, CAR = cardiac, HEM = hematologic. Intensity of grey indicates the discriminating power of input variables. White background indicates no discriminating power of the listed variables. CAR and HEM have no influence on the classification. Two clusters, marked 1 and 2 in the first line of the table (Cluster Label), were found in this preliminary run. (**B**) Graphic output of the second run of two-step cluster analysis based on only 4 of the 6 organ dysfunctions (*n* = 101; CAR and HEM were kept out), displaying the discriminating power of each organ dysfunction as odds ratio. For abbreviations, see (**A**,**C**). (**C**) Graphic output of the second run of two-step cluster analysis based on only 4 organ dysfunctions (*n* = 101, CAR and HEM not in use), displaying cluster quality as good, based on cohesion of cases within clusters and separation of clusters. Six well-defined clusters, plus one cluster with cases that could not be classified (“miscellaneous” or “−1”), were identified. For abbreviations, see (**A**).

Subsequently, a two-step cluster analysis was run using SPSS 21 software in order to discriminate sampled patients (*n* = 101) with severe malaria according to the presence or absence of the six organic disorders used to estimate the SOFA score. Figure 5A shows the two homogeneous clusters obtained on the first run, indicating that two of the organic disorders, cardiac and hematological dysfunction, did not present discriminatory power in the studied group. In addition, a second run of two-step cluster analysis (*n* = 101) was performed using only the four organic dysfunctions detected as having discriminant power (excluding the two organic disorders CAR and HEM that showed no discriminating power in the first run) in order to reduce the background noise of the previous analysis. Thus, Figure 5B shows the discriminating power of the four organ disorders (renal, neurological, cardiac, and ARDS), quantified as odds ratios, which detected six homogeneous clusters and one cluster with atypical behavior that included cases that could not be classified in the six main clusters. Figure 5C demonstrates the “good” quality of the aggregation of cases in clusters in terms of both the cohesion of cases within each cluster and separation between clusters. It is thus demonstrated that the sample is not homogeneous and that further analysis of mortality should consider this information.

**Figure 6:**
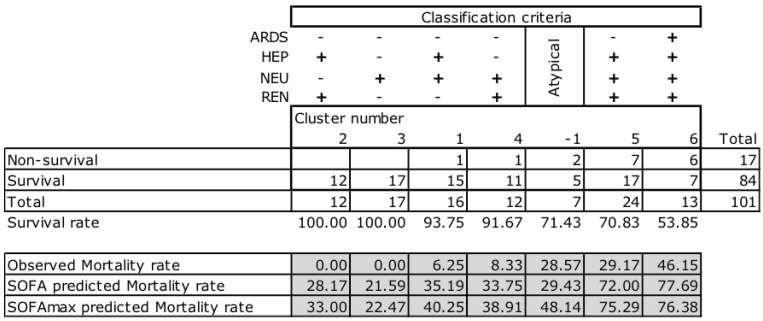
Classification criteria of the 6 + 1 clusters, and mortality distribution within each cluster. Survival and mortality rates are shown in % of total cases observed (*n* = 101). Each column represents a numbered cluster (1–6) and one atypical cluster numbered by the software as −1. The classification criteria defining each cluster is indicated at the top of each column/cluster for the 4 different organ dysfunctions (*n* = 101; CAR and HEM not in use) that are relevant.

Figure 6 shows the classification criteria for 6 + 1 clusters detected based on the presence or absence of four organic disorders (ARDS, HEP, NEU, REN) observed in patients with severe malaria. It is observed that the seventeen fatalities are included in groups with two or more of the four discriminating organ disorders, with neurological dysfunction present in all cases. However, in Cluster 3, consisting of seventeen patients admitted with neurological dysfunction associated with anemia or thrombocytopenia, there were no fatalities. Additionally, in Cluster 2 (*n* = 12), which includes patients without neurological dysfunction, there were also no fatalities.

The lower part of Figure 6 shows observed relative mortality, compared to the number of expected/predicted relative mortality by the two variants of the SOFA score: mean SOFA and SOFAmax. In the six typical groups, the expected mortality is much higher than the observed mortality. It is also observed that the number of expected relative mortality, predicted by the average of SOFA and SOFAmax, is very similar. In addition, the “atypical cluster” group, identified as “−1”, is the only group where we observe (i) a considerable difference (approximately double) between the two expected mortalities predicted by the two variants of SOFA, and (ii) a mortality rate that is very similar to the expected mortality predicted by the average SOFA. In general, the relative case mortality observed in patients with severe malaria is much lower than expected (χ^2^, *p* <0.001), and patients with a higher number of organic disorders (Clusters 5 and 6) had a lower than expected number of fatalities (χ^2^, *p* <0.001).

## 4. Discussion

The age distribution of the patients enrolled in the present study is characterized by the fact that the admitting ICU, despite not being a pediatric unit, accepts young patients (≥10 years of age). In fact, more than 57 of the patients enrolled were aged below 20 years; the median was 16 years, and there were no patients above 57 years. This may have influenced the lack of association between age and the outcome (χ^2^, *p* = 0.398) that we found.

Studies with severe malaria patients showed a significant correlation between age and probability of death [7,8], where the mortality increased steadily from 6% among patients aged <10 years to 36.5% among patients aged >50 years. Gender (with male predominance) did not influence the outcome (χ^2^, *p* = 0.884) in the present study. However, the number of female patients above 40 years in the study sample was too small to confirm the results from Kandanga and colleagues [9], indicating that in *P. falciparum* malaria, women above 40 years have higher mortality than younger women and men of the same age.

Local native patients were 87% of the total, with prior malaria infection history. Only patients more than 9 years old were included in this study. These patients have, in theory, acquired protective malaria immunity. Only 10% were considered nonimmune (no stay in Africa over the previous 6 consecutive months and no previous malaria episodes). This epidemiologic characteristic had no influence over the outcome (χ^2^, *p* = 0.620). Another published study confirms these findings. Marks and colleagues studied 124 patients with imported *P. falciparum* malaria in England and did not find a correlation between the severe clinical features observed and the immunity state by birthplace and residence of the patients [10]. This probably reflects the labile and incomplete characteristics of immunity against *P. falciparum*, which needs multiple and continuous exposures to species-specific parasitic antigens [11].

Concomitant medical conditions (comorbidity) were present in 23.7% of the patients, a finding consistent with a young population, mostly without a background of chronic pathologies, and had no influence on the outcome (χ^2^, *p* = 0.14). Even the low immunity observed in patients that were HIV-positive (3 patients, all with a CD4 cell count of less than 500/μL) did not influence the outcome (χ^2^, *p* = 0.62). There is no consensus on this subject in the literature [12,13,14].

In the present study, there was no statistically significant difference between average parasite density (parasite per μL) in blood smears vis-a-vis the outcome (one-way ANOVA, *p* = 0.923). Contrary to the results in the present study, in Malaysia, a study showed that the parasite density was globally higher in more critical patients and nonsurviving patients [15]. Phillips and colleagues studied 482 patients with severe *P. falciparum* malaria and found a significant difference between parasite counts greater than 100,000 per μL (2%) and the severity of the disease. Furthermore, a multivariate analysis showed that the existence of more than 2% of parasitized erythrocytes aggravated by five times the clinical condition [16]. However, Achidi and colleagues [17], in Cameroon, did not find a correlation between the parasite density and the severity of the disease. This study included 908 children with *P. falciparum* malaria, with both complicated and noncomplicated disease; the authors suggested that the parasite density is not a significant factor influencing the severity of the clinical condition of the infection. The correlation between the most severe clinical features of malaria and parasite sequestration in capillaries and venules, and rosette formation in several organs, can explain the low peripheral blood parasite count, particularly in cerebral malaria [18,19]. Based on these publications and the findings of the present study, it can be suggested that, especially in an endemic context, a low peripheral blood parasitic load alone should not invalidate the diagnosis of severe malaria and should not be used as an isolated prognostic marker.

Multiple organ failure is the leading cause of morbidity and mortality in ICUs. An observational prospective study in 79 ICUs (a large database of 7615 patients) concluded that critical patients over 60 years, showing a SOFA score above 10 points and a positive or unchanged 5-day trend, had a mortality of 100% [6]. The correlation between the degree of organ failure and the mortality estimated by SOFA score is well established in severe sepsis patients [20]. Furthermore, the number of simultaneous organ dysfunctions has a negative correlation with survival. Patients with four or more organ dysfunctions, with a SOFA score greater than 10, have a probability of death greater than 50% according to the SOFA calculator (http://clincalc.com/icumortality/sofa.aspx). In the present study, 39% of the patients had four or more organ dysfunctions and an observed 46.1% mortality rate (see Figure 4). Furthermore, survivors had a significantly lower average number of dysfunctions than nonsurvivors (2.99 ± 1.07 versus 4.24 ± 0.83, respectively; *p* < 0.001; see Table 4). The present study confirms the usefulness of the number of simultaneous organ dysfunctions in determining the prognosis of severe malaria patients.

The total number of life support interventions between the two outcome groups was significantly higher in surviving patients (χ^2^, *p* < 0.001, see Table 4). This difference suggests that the timely use of vital support techniques had a positive impact on the outcome, particularly in patients with neurologic and renal dysfunctions, where the early decision to ventilate and begin hemodialysis saved patients’ lives. Early identification of renal dysfunction (monitored according to RIFFLE and AKIN criteria) had a definitive impact on patient survival, as previously suggested by Trang and colleagues [21].

The average SOFA score in patients of the present study was 8.92 ± 3.2. The statistical analysis of all variables used to generate the SOFA score showed a significant difference (χ^2^, *p* < 0. 001) between the surviving and nonsurviving groups of patients (see Table 4). ROC curves show similar and significant AUCs (>0.50) for average SOFA and SOFAmax scores, showing that these indicators have enough sensitivity to correctly predict the initially expected outcome (before intervention in ICUs can produce results). However, the observed fatality rate (16.8%) was much lower than the one estimated by SOFA upon admission to the ICU (>50% in 64.3% of the patients), suggesting that this score, although efficient in the initial daily and sequential assessment/management of organ failure in patients with severe malaria, has a low specificity and is not effective for correctly establishing the final prognosis [14].

Cerebral malaria can be an isolated organ dysfunction, or it may be included in multiorgan dysfunction. It is consensual that cerebral malaria is the main cause of death in malaria regardless of the patient´s age [7]. However, in the present study, all the patients (*n* = 17) with only cerebral malaria as a discriminant organ dysfunction and major admitting criterion to the ICU (but with nondiscriminant hematological dysfunction) survived. The very early decision in favor of immediate endotracheal intubation and ventilator support in unresponsive coma (Glasgow ≤7) patients may have avoided severe intracranial hypertension, irreversible brain swelling, and subsequent brain death.

The two runs of the “two-step cluster analysis” algorithm (SPSS 21) identified six homogeneous clusters (subgroups of patients) and one “atypical” cluster, in which four organ dysfunctions (respiratory, hepatic, renal, and neurological) were judged as significant discriminants in the present study. In the six homogeneous clusters, predicted mortality correlated well with observed mortality, and the data obtained confirmed that higher mortality in a cluster is associated with a higher number of discriminant organ dysfunctions present. Neurologic dysfunction (cerebral malaria) was associated with all death cases, but only when combined with other dysfunctions (Figure 6). Alone, neurologic dysfunction was associated with a 100% survival rate (Figure 6, Cluster 3).

## 5. Conclusions

This observational and prospective study describes a sample (*n* = 101) of a young African population, living in a stable intermediate-malaria-endemic area (Luanda, Angola), admitted with severe malaria into an ICU. Gender, age, peripheral blood parasite load, and epidemiologically defined immunity against malaria did not significantly influence the outcome. The patients had very severe disease, as indicated by the high number of organ dysfunctions frequently present. Early life-support interventions increased the chances of survival, as indicated by the higher number of interventions in the surviving group.

The SOFA score showed good discriminating power for monitoring severity but was not effective in correctly predicting the prognosis in severe malaria: the observed fatality rate was lower than the one predicted/expected by average SOFA or SOFAmax. The “two-step cluster analysis” algorithm allowed the identification of six homogeneous clusters of patients that displayed several specific patterns/combinations of respiratory, hepatic, renal, and neurological organ dysfunctions. Within each cluster proposed by the algorithm, mortality could be better predicted. Based on the preliminary results generated by the present study, it is expected that “cluster analysis” may, in the future, refine or improve the prognosis based on acute organ dysfunction in severe malaria cases.

## Figures and Tables

**Table 1:** Parasitic density of *Plasmodium* sp. in blood smears of individuals diagnosed with malaria.

Parasite Density	Patient Count	%
Negative	08	7.9
≤50,000/µL	58	57.4
˃50,000 ≤100,000/µL	14	13.8
˃100,000 ≤150,000/µL	01	0.9
˃150,000 ≤200,000/µL	01	0.9
˃200,000 ≤250,000/µL	18	17.8
No information	01	0.9
TOTAL	101	100.0

**Table 2:** ANOVA analysis of laboratory data of patients with severe malaria admitted to the ICU of a hospital in Luanda, Angola.

Laboratory Data	Surviving (*n* = 84)	Non-Surviving (*n* = 17)	*p*-Value
Hemoglobin (g/dL)	8.30 ± 2.70	6.80 ± 2.31	0.045
**Platelet count (uL) ^1^**	**89.30 ± 99.90**	**55.30 ± 42.20**	**0.174**
Blood urea (mg/dL)	102.61 ± 91.44	187.47 ± 136.09	0.002
Serum creatinine (mg/dL)	2.53 ± 2.29	4.58 ± 3.38	0.003
**Serum total bilirubin (mg/dL) ^1^**	**5.07 ± 7.35**	**6.59 ± 6.06**	**0.424**
Serum AST (UI)	71.80 ± 69.30	156.50 ± 187.40	0.003
Serum ALT (UI)	119.90 ± 122.00	254.70 ± 210.10	0.006

^1^ Nonsignificant difference is shown in bold characters.

**Table 3:** Life-support techniques used in hospitalized patients with severe malaria.

Life-Support Interventions	Number of Patients	Percentage
Vasoactive treatment (VT)	01	0.90
Hemodialysis (HA)	15	14.80
Invasive ventilation (IV)	11	10.80
VT + IV	03	2.90
VT + HA	01	0.90
HA + IV	09	8.90
VT + HA + IV	01	0.90
No vital support	60	59.40
TOTAL	101	100

**Table 4:** Variables related to severity of clinical status and life support interventions for individuals hospitalized with severe malaria. Some patients received multiple life-support interventions.

Variables	Surviving (*n* = 84)	Non-Surviving (*n* = 17)	*p*-Value
Average Glasgow score	10.31 ± 3.14	7.47 ± 3.5	0.001
Average SOFA score	8.1 ± 3.24	12.94 ± 3.98	<0.001
Average number of patient dysfunctions	2.99 ± 1.07	4.24 ± 0.83	<0.001
Number of patients with respiratory distress	07	08	<0.001
Number of life support interventions	31	25	<0.001
**Number of patients with amine-usage support ^1^**	**03**	**01**	**0.656**

^1^ Nonsignificant difference is shown in bold characters.
